# Not Quite Enough: How the Pandemic Failed to Save Europe: Luuk van Middelaar, *Pandemonium: Saving Europe*Agenda Publishing, 2021, 199 pp. ISBN:978-1788214230

**DOI:** 10.1007/s12115-022-00777-x

**Published:** 2022-09-28

**Authors:** Wolfgang Streeck

**Affiliations:** Max Planck Institute for the Study of Societies, Cologne, Germany

Luuk van Middelaar’s most recent book on Europe, like his previous work, is serious stuff.^1^ Don’t expect your run-of-the-mill “European integration” spiel, liberally funded by the European Commission, dealing with issues like How-the-Commission-constructed-a-Treaty-base-where-there-is-none; or the encouraging results of the latest “European Semester” and what additional data Croatia must supply next time for even more economic stability and convergence to ensue; or why monetary union requires fiscal union to deliver its full benefits; and how the Treaties must be rewritten to consummate the unity of Europe by allowing for the magic of neo-functionalist spillover. None of the usual obsession here with the design and implementation of “programs”, their odds and ends and how they grow out of the infighting between the Commission’s General Directorates, the EU’s various supranational would-be authorities and its member states — all of this on the assumption that “integration” must ultimately move forward as foreseen by “integration theory”.

Like his previous work, van Middelaar’s book is not about functional constraints — what the Germans call *Sachzwänge* — but about politics writ large. Indeed he would probably not object if we defined what he means in a Carl Schmittian sense: the forging and defending of “communities of fate” in moments of existential threat, by strong leaders understanding and exploiting the need for extraordinary action in extraordinary moments, producing the right “narratives” — van Middelaar’s favorite concept — for the political theatre that is the public sphere, a dramatic-sentimental rhetoric of fear and hope (pp. 113, passim). For a conceptual scaffold, van Middelaar relies on a distinction between “rule-politics” and “event-politics”, the former not really deserving of the label politics, the latter “revolv[ing] around our collective dealings with contingency, our joint approach to the vicissitudes of fate.” (p. 15) To the extent that it is what it claims to be, European integration must therefore involve progress from “a structure devoted purely to ‘rule-politics’”, as embodied in yesterday’s European Community and cultivated by the Brussels institutions operating the “Community method”, “to a set-up also capable of ‘event-politics’” (p. 21), associated for van Middelaar with the European Union post-Maastricht, developed and further developing beyond the setting, administering and obeying the forever growing body of rules forming the EU’s elephantine *acquis communautaire.*

What is needed for such progress, according to van Middelaar, are crises that pose a lethal threat to “Europe” as a whole, forcing the *community* of European states to realize that it must become a *union* in order to survive. Conflicts among member states do not really qualify, since they can and must be settled by the politics of rules. Integration in the sense of union-building is driven by challenges from the outside, pushing Europe “further along the path towards a community of destiny that addresses events as a unified whole” (p. 15) — challenges from others, from *Feinde*, or foes, in a generic sense, that behave unpredictably because they follow different rules, making it impossible to deal with them under your own rules. Event-politics is the politics of *Notstand*, which in turn is *die Stunde der Exekutive*, the hour of the executive, not of the bureaucrat, the law-maker or the judge, when “necessity knows no law.” (p. 21) While in the Community world “politicization” of European integration is a threat as it may wake up the sleeping dogs of political passions, interfering with the technical making of efficient rules, for van Middelaar’s Europe, it is the indispensable driving force for it becoming not just integrated but unified, a political entity with a will to power. Indeed, those intent on building a state must know if not how to fabricate a crisis, then how to exploit one, turning “the political deployment of controlled panic” into an “instrument of governance” (p. 16), if needed by making the existential threat confronting their community appear even more existentially menacing.

Political writers write from their own perspectives, and it should not be deemed inappropriate to consider their personal experience and the social position from which they look at the world. van Middelaar, a Belgian historian, now a professor at both the University of Leiden and the Catholic University of Louvain, has served in various staff positions at the European Union. From 2009 to 2014, he was a member of the cabinet of Herman van Rompuy, the first full-time president of the European Council, where he worked among other things as a speechwriter. What he saw of the European Union was the exclusive circle of heads of state and government, 27 of them, led by a president who need not be but always is a former member of their club. From there, the pinnacle of EU power and prestige, the European Commission, guardian of the Community method, appears as a subordinate secretariat obsessed with pedestrian legality and efficiency, a grey bureaucracy compared to the glamour projected by the half-yearly assemblies of the national leaders of Europe, and in times of crisis by any number of dramatic emergency meetings.

Politics, in van Middelaar’s world, is high politics, the politics of states vis-a-vis states, not the low politics of conflicts between groups, or classes, within societies organized by states. In high politics, domestic conflicts, including those deriving from the contradictions of capitalism, are for the men and women of state to keep under control, so as to not interfere with the unity of the nation — or in Europe: of the Union of European states — in its relations with the outside world. High politics of the van Middelaar variety presents itself to the public in what colloquial German calls *Haupt- und Staatsaktionen*: in the carefully staged pomp and circumstance of European summit meetings, where national leaders meet in full ceremonial display of the symbolic trappings of their power; it is elitist to its core. Ordinary citizens may be experts on their private lives and special interests, but they know little of their nation’s interests in its relations with other nations, on their nation’s and their state’s historical mission. Given this deplorable inability to look beyond the narrow confines of everyday life, it is inevitable and entirely justified for state elites to deploy all sorts of simple, well, “narratives”, sentimental and emotional, for the good purpose of national political education, so as to overlay the trivial concerns of the people in the streets with the non-ordinary, extra-ordinary concerns of nations and their leaders — with Max Weber, the *außeralltägliche* (extraordinary) concerns of history — a little exaggeration included if it serves the purpose of generating “solidarity” and pushing divisive everyday concerns, such as class interests, off the stage of the political theatre.

Now on to the virus, and the way it allegedly advanced the famous “ever closer union of the peoples of Europe”. For van Middelaar, the pandemic was the sort of event that, when used well, and well-used it eventually was, allows leaders with the right kind of political instincts to do what they are always waiting to do: to get citizens to sacrifice some of their trivial everyday interests for the higher purpose of collective self-assertion. With COVID-19, a life-threatening enemy appeared on the scene that follows its own logic, natural, it is true, not social, but identical in its consequence: the need to unite in order to push it back, indeed root it out. For European event-politics waiting for its state-building *kairós*, for a Europe needing to “experience itself as a body politic” (p. 20), the virus seemed to deliver an ideal occasion for the kind of rhetoric of fear, hope, and salvation that, in van Middelaar’s world, creates nations as historical actors, as deeply felt communities of destiny — the pandemic, as was often heard, as a moral equivalent of war.

Without much ado, van Middelaar admits that with respect to public health in a narrow sense, the EU failed spectacularly (pp. 53ff). Attempts, especially by the Commission, to manufacture a narrative of technocratic expertise and efficiency are coolly dismissed. As it turned out, no preparations had been made in the realm of rules-politics for a critical event of the COVID-19 kind: no masks, no protective clothing, no action plans. Soon the “vaccine wars” (p. 93) ensued: who was to order what and how much and from whom, at what price? van Middelaar estimates that inoculation was delayed in EU countries by about two months as a result of bureaucratic blundering (p. 103) — no figure is suggested, though, for the number of avoidable deaths that ensued. Single-mindedly focused on protecting “free movement” and the Schengen rulebook, the Commission insisted that national borders be kept open while member states, protecting their citizens instead of the rules, closed them anyway.^2^

For van Middelaar, Brussels mismanagement does not come as a surprise, also because public health is none of the EU’s business anyway, according to the Treaties. More importantly, however, in van Middelaar’s idea of Europe, nation-states continue to be essential, quite in contrast to standard “integration theory”, partly because they are closer to the situation and the needs of their citizens, but mainly because they are uniquely capable of the kind of rulebook-transcending event-politics needed in critical moments. As van Middelaar states: “The unyielding faith that the ‘true Europe’ must be built in defiance of the member states, rather than with then, feeds public skepticism and stands in the way of the development of a joint capacity to act.” (p. 40).

One may wonder, in this spirit, what van Middelaar thinks of the budget rules of the European Monetary Union (EMU) that over a decade and more forced a country like Italy into austerity, to cut back on government spending, among other things, on health care, for the sacred sake of a balanced primary budget. And how does the abject failure, not just of the EU but also of European nation-states, to make a European voice heard in relation to the WHO and its contingency planning in the field of “bio-security” fit in the picture, including the strict avoidance on the part of European leaders of the still wide-open question of Chinese-American collaboration on “gain-of-function” research with corona viruses, in laboratories like that at Wuhan.

Nevertheless, in sum, van Middelaar considers the pandemic a decisive moment in what in the title of a previous book he had called “the passage to Europe”. That it became such a moment was owed to the way in which European leaders seized on the Europe-wide compassion with the suffering, especially of Italy, as supposedly documented by the ever-present “pictures from Bergamo”, to move European state-building to a new level. In an enthusiastic account with a sometimes kitschy flavor, van Middelaar presents a “narrative” of the origin, in the early summer of 2020, of the so-called Corona Recovery Fund — which, according to Ursula von der Leyen, the current President of the European Commission, never at a loss for a fancy neologism, remade the EU into a NextGenerationEU (NGEU), written as one word. Dramatis personae include the foxy Charles Michel, Council president at the time, and the smart Emmanuel Macron, but there are also bad guys, like German economics professors who know no economics, and not to be forgotten the thrifty Dutch. The main role, however, van Middelaar reserves to Angela Merkel, who in a veritable panegyric is claimed to have suddenly understood during the battle against the virus that she had to seize on the passion of the moment and become “Lady Europe”, emancipate herself from the miserly economics of the Swabian housewife, and allow the Union to set up a credit-financed emergency fund of €750 billion. €390 billion would be grants, the rest loans, with the lion’s share of the total, €209 billion altogether, going to post-Bergamo Italy.

In a true panegyric, there is no room for shades of grey; Roman emperors all-too-easily took offense. No mention, therefore, of the fact that in order to event-politically transgress the prohibition of the Treaties on borrowing by the EU, a legal construction outside of the Treaties was needed that required a unanimous agreement of all 27 states. These included Hungary and Poland, which in return had to be assured by von der Leyen that the EU’s rule-of-law proceedings against them would be suppressed — a promise on which she has yet to make good. Further, for all member states to feel sufficiently solidary, each had to be given a share in the booty, even Germany (€28.8 billion) and France (€38.8; figures refer to grants only, as adjusted in June 2022). For this, the Commission had to cook up a formula that had little to do with COVID, as some member countries had not been much affected by it; what mattered was that Italy (and Spain) came out in front (Italy €127.7 billion, €81.8 of which as grants, Spain €140.4 billion, of which €77.3 as grants). Moreover, not to overdo the solidarity, member countries were made liable for the common debt only in proportion to the size of their economies, rather than jointly for the debt as a whole, thereby avoiding the €750 billion becoming in effect so-called Eurobonds. (All in all, the grant component of the fund, which is the one that matters, may be estimated to amount to at most 0.5 percent of the EU’s GDP over four years a quite limited sacrifice for a dying neighbor.) What was and had to be left open was how the solidarity-debt would be serviced and repaid and by whom, with respect to both member states borrowing from the Union and the Union, or the collectivity of its member states, borrowing from the capital markets.

Even more importantly, where van Middelaar celebrates Merkel’s “leap” into a great European tomorrow (p. 80 ff.), he forgets to mention that since the fiscal crisis of 2008 at the latest, it was probably the foremost goal of German policy in Europe to keep Italy in the EMU, regardless of a deep mismatch between its rules and the contingencies of Italian capitalism. All German parties were and continue to be of the view that an Italexit in whatever form would be a disaster for a German economy whose prosperity depends on the euro, and in part on the steep gradient of prosperity in the euro area between its north-western center and its Mediterranean periphery. To prevent Italy from leaving — more precisely, to prevent Italy’s volatile political system producing an “anti-European” government — Germany under Merkel had always been willing to pay any price, as long as it could be hidden from the German public and somehow sold to the German Constitutional Court. What had changed in 2020 was that after “Bergamo”, a greater effort than usual was needed to keep the Italian public and its politics euro-friendly, by offering the Italian political class a “narrative” to the effect that this time, unlike in the past, the economic decline of Italy would be effectively reversed by “Europe”. While the same had been promised before, it now came with a public fanfare and a pan-European symbolism that was new, and perhaps this was all that was new. For van Middelaar, of course, this was new enough, given that in his kind of politics, what matters are not economic interests, or social structures of advantage and disadvantage, but telling the right story, the effective narrative of a politics of emergency ready to be staged in the theatre of contemporary politics.

There is another hero in van Middelaar’s account of the Great Breakthrough of 2020, in the same league as Merkel, by the name of Mario Draghi, then recently retired as President of the European Central Bank. van Middelaar can barely contain himself when recounting how “Super Mario”, as he does not refrain from calling him (p. 110), “saved the euro”, and with-it “Europe”, just by saying a magic few words at the right magic moment, breaking out of the straightjacket of rules-politics and thereby proving himself to be the kind of leader that makes the decisive difference. To van Middelaar’s elation, when the sitting Italian government was unable in 2021 to agree on a proper allocation of their country’s share in the Recovery Fund, the President of Italy, undoubtedly with encouragement from Berlin and Paris, “was prompted to … bring out the country’s best horse from the stables, … Mario Draghi, to form a cabinet.” (p. 108 f.). The result was the grandest possible Grand Coalition, an almost-all-party government, promising an end of politics as Italy knew it, so that “the man many see as having saved the euro in 2012 [could] now save Italy” (p. 109) with the support of united European solidarity. Italy saved, the euro saved, Europe saved, thanks to Merkel and Draghi.

Van Middelaar’s book ends with a chapter entitled “Geopolitics: between China and the United States”. In it, van Middelaar further develops his version of “the uniting of Europe” (the title of Ernst Haas’ seminal book from 1958 that laid the foundation for the neo-functionalist theory of “European integration”) by placing it in the context of what at the time of his writing, roughly coincident with Biden’s accession to the US presidency in early 2021, presented itself as a new stage in an evolving geopolitical conflict between the USA and China. Here, too, it is the pandemonium of the pandemic that serves as an effective driver of European state formation. By intensifying the conflict between the declining global power of the USA and its rising rival, China, the pandemic is claimed to have opened a geopolitical space for Europe that it is forced to fill by whipping itself into political shape, or else be condemned to eternal dependence and irrelevance.

In making his case, van Middelaar points to a remarkable complementarity between China and the USA. Like its divisive face-mask diplomacy, China’s refusal to cooperate with the West in its search for the origin of the virus aimed at laying bare the failure of the European Union to prevent and contain the pandemic. More than ever, this recalled the Chinese effort to penetrate the European geopolitical space with its New Silk Road project, intended to make countries on the European periphery dependent on Chinese trade and credit while exploiting European free markets for advanced technology. At the same time, the USA seemed to be entirely lacking the kind of strategic determination mustered by Chinese nationalist capitalism, certainly as far as Europe was concerned. In fact, under Trump, it appeared to have finally turned both inward — “America first!” — and unpredictable, and in any case no longer able to underwrite the “liberal international order” that it had promised after the fall of the Soviet Union in the early 1990s. Moreover, by accusing China of having allowed the “Chinese virus” to escape into the world-at-large, Trump, deliberately it seemed, raised tensions between the USA and China, perhaps in preparation for a military confrontation. It is against this background that van Middelaar calls upon “Europe” finally to develop what he, with Macron, calls “strategic autonomy” (p. 155) — to avoid, as he puts it in two chapter subheadings, being “Colonized by China” (p. 156) in a new era “After the Pax Americana” (p. 165).

To his credit, van Middelaar does not shy away from the radical implications of his third way vision of a united Europe more or less equidistant from China, with its determined *Wille zur Macht* (Will to Power) and the USA, with its unsustainable civilizational, political, and economic universalism, which in fact is nothing else than nationalist imperialism on the part of a national society in decline. Squeezed between the two, Europe needs, van Middelaar suggests, as we might by now expect him to, a “story” of its own, different from the Chinese and the American stories — “narrative sovereignty”, as he puts it. This, however, cannot be achieved “without strategic autonomy” (p. 155), which in turn requires that Europe as a Union learns to “engage in event-politics, as a player with skin in the game, with power and a narrative. In respect of both”, the pandemic is said to have been “an historic turning point” (p. 156), leading to the recognition that in a world “where not everyone … has to become the same”, a “self-image that claims to be based on universal values” is no longer enough (p. 164; see also, for a trenchant critical assessment of universalism, pp. 27–33).

Unlike other “pro-Europeans”, van Middelaar is adamant that Europe’s future strategic priority must be autonomy, not just from China, but also from the USA. Clearly, he is, or was when the book was written, far from being an “Atlanticist”. For an initial impression of the new US president, van Middelaar notes that Biden, more than both presidents preceding him, “is playing the card of American imperialism”, trying to assemble as many countries as possible in an alliance with the USA in its upcoming struggle with China, “positioning the U.S. again as the self-conscious leader of the free world” (p. 168). This, however, will not work anymore: “The days of global supremacy are now out of reach to both those great imperial powers”. This, van Middelaar continues, “creates a need for forms of power balance and coexistence – and hence thinking in terms of pluralism”, which makes it necessary for Europe in particular “to promote a multipolar order”. As a precondition, “the Union must first develop the ambition to be a relevant pole itself, a power among powers” (p. 170), for which it must learn to resist “the lure of universalism” — “the temptation to be absorbed in a ‘West’ that includes the U.S. and Canada…, although the compelling effect of the narrative machine that is Washington and Hollywood in creating a new [common] enemy should not be underestimated” (pp. 171–2). van Middelaar concludes by demanding “a strategic conversation at the highest political level” which would “force a continent that after 1945 escaped from the morass of self-destruction by mentally clinging to universal values, borderless space and abstract time to re-engage with geography and history in the full sense of the word.” (p. 172 f.)

As this review is being written, roughly two years since van Middelaar finished his book, two new “events” have occurred, overlapping and not entirely unrelated: the collapse of the NGEU recovery plan and the outbreak of the Ukrainian war. When he wrote van Middelaar had yet to witness the downfall of his hero, Mario Draghi, as Prime Minister of Italy, and with it the utter failure of the Recovery Fund not just to save Italy but also to generate a lasting van Middelaarian “narrative” of European solidarity to be staged in the European public-political theatre. This time, when it was needed, paraphrasing Draghi in his previous incorporation, “it was not enough”. After a little more than a year in office, “Super Mario” resigned in July 2022, requiring fresh elections to be called for September of that year. In the ensuing campaign and the European politics surrounding it, the €209 billion Italy had been promised in all play no role whatsoever. This is not surprising, except perhaps for true-believing professional producers of up-beat state-building Euro prophecies designed somehow to become self-fulfilling. Not only has it sunk in that the money Italy is supposed to receive is no more than a drop in the ocean compared to the country’s economic problems. (By mid-April 2022, two years after the fund had been set up, the Commission had paid Italy just 24 percent of what it is to receive, €46 bn, which amounted to less than 15 percent of Italy’s combined public deficit in 2020 and 2021, or just 1.9 percent of Italian GDP in 2021.) Experience with the Fund also confirmed that Italy’s problem was not primarily a lack of cash, or credit. More important was and continues to be the absence of a political and administrative infrastructure capable of planning and conducting a large-scale public investment offensive, and generally of organizing and sustaining an efficient capitalist economy under the constraints of monetary union with the countries of North-Western Europe. By the middle of 2022, with Draghi already a lame duck, his government, just as its predecessor, had yet to come up with a credible plan for how to spend the Recovery money so that it would raise the growth potential of the Italian economy, not just in a “narrative” but in fact.

Then, in the early months of 2022, with Draghi still in office but already a spent force, came the Russian invasion of Ukraine, which almost immediately turned van Middelaar’s “pandemonium” into a pale memory from a long-distant past. If ever postwar Europe was confronted with a life-and-death “event” allowing, if not calling for, extraordinary rather than ordinary politics, then it was and continues to be the Ukrainian war, and not just after the Russian troops went in but already in the long winter months leading up to the invasion. Where was “Europe”, van Middelaar’s up-and-coming political superstate whose governors, according to him, had in the pandemic so valiantly crossed the border between rule-politics and event-politics, riding high on a wave of Europe-wide solidarity, casting aside the rigid prohibitions of the Treaty and, in installing Draghi-the-miracle-man as viceroy of a true union that seemed finally to have grown out of its Community past?

In fact, it was not the EU that acted for “Europe” in the hour of truth but the EU’s Big Sister, Europe’s transatlantic extension, NATO, including the USA in addition to a number of not-so-united European states — an alliance governed, not by 27 heads of government, or by any one of the many presidents the EU allows itself, but by POTUS himself, and his military planners at the CIA, the Pentagon, the Joint Chiefs of Staff, the National Security Council, and similar places that we may not even know. As to the EU, it was and remained once again reduced to displaying its political insignificance and technocratic ineptitude. With her usual hype, the President of the Commission, von der Leyen, promised to organize the most effective sanctions on Russia in the history of mankind, designed to “peel off one layer after another of Russia’s industrial economy”, only to be told that rhetoric of this sort might be considered evidence of the EU having become a party to the war. The promise was never repeated. It also turned out that the sanctions thrashed out by the Commission on American order misfired dramatically, threatening to cause a severe energy shortage in Western Europe during the winter of 2022/23 and perhaps beyond, as well as a food shortage in far-away places around the globe. Moreover, attempts to mobilize another round of European “solidarity”, this time in the form of EU-wide energy sharing, failed as well, among other things because the nuclear culture of France and the anti-nuclear and pro-gas-cum-wind-and-sunshine culture of Germany proved as incompatible as could have been expected.

As a side show, Ukraine, together with Moldova, was promised speedy admission to membership, with von der Leyen, dressed in the blue and yellow colors of the Ukrainian flag, declaring that, by fighting and suffering on behalf of “all of us”, Ukrainians had “deserved” to become official members of the “European family”. Later she must have been reminded that her organization has complex admission procedures and demanding admission criteria, and that there was a long line of applicants, on the West Balkans for example, who would ask to be dealt with on a first come, first served basis. (More difficulties arose when Chancellor Olaf Scholz, certainly with French assent, made further enlargement conditional on institutional reforms, for example a reduction of the number of Commission members.) Meanwhile member states, even disregarding notoriously rebellious Hungary, could and would no longer hide that they were far from unanimously behind the Commission’s unmitigated commitment to the Ukrainian government’s war aims. While Poland, the long-time state enemy number one of the Brussels establishment, took a position as militant as that of the USA and of von der Leyen, France at the other end of the spectrum advocates a settlement that allows Russia to “save face” (Macron). Germany, as usual, is somewhere in between, helping itself with the long-effective Merkel method, which is to agree to whatever can at the moment be agreed by all, without necessarily intending to follow up on it. For example, while von der Leyen, with the public assent of the European Council, promises loans to Ukraine in the order of endless billions of euros, member states, in the privacy of their capitals, have to come to terms with the fact that such loans will never be paid back. Unable to say so for political reasons, they conveniently fall back on their time-tested rule-politics, resulting in the Ukrainian government publicly complaining a year later that not a single euro had yet been paid out. Especially for a country like Germany, discovering constitutional impediments to letting the EU borrow on behalf of non-EU countries is not difficult. The same sort of excuse offers itself as long as Germany, like other member states and indeed the EU as a whole, remains cut off from decisions on “the West’s” Ukrainian war aims — decisions made, if not in Washington, then in Brussels, although not at the EU but at the, conveniently closely located, headquarters of NATO.

So, did the Ukrainian war entail a second, or perhaps a first or even the last chance for a magic European political moment of the van Middelaar kind, after the disaster of the pandemic and the failure of the recovery fund to save Italy in order to save Europe? The sobering answer is that there may indeed have been a window for an autonomous European event-politics, in those months and weeks when it might still have been possible to let the Americans and their Ukrainian friends know that Europe wanted not a war but a settlement, if necessary just a preliminary one, along the lines of the Minsk accords, with Europe in the driver’s seat, rather than far-away USA. If ever there was a critical moment calling for an improvised collective response of the Western European continent in the interest of Europe as a whole, a moment for united power-making and power-wielding, this might have been it, not to be repeated for a long time to come. Having failed to live up to the challenge of peace-making on the Eurasian continent, Western Europe may, in a formative period in global politics, be in the hands of the USA, reduced to a military and economic auxiliary in its upcoming war with China — hands that in recent decades have proven both fickle and clumsy, lacking intelligence or purpose or both, and as a result all-too-easily given to violence.

The good news, if there is any at all, may be that, as easily as the USA gets into a mess, it tends to lose interest in it and withdraw to their continent-sized remote island. US opinion may at some point resolve that it can no longer afford two wars at the same time — another “America first” moment. Corruption in Ukraine — where are all the guns, where is all the money we sent them? — may serve as a pretense for withdrawal. Also, Russia’s emerging alliances with Iran, India, and even Turkey, a NATO member after all, on top of its rapprochement with China, may make US attempts to subject the country to something like a Morgenthau Plan appear futile even to the most adventurous pursuers of global causes in the White House and its environs. Faster than they came the Americans may go, leaving huge chaos to be dealt with by the locals. One indication that there is a serious dispute in the US political establishment on its role in the Ukrainian war may be that strange trip of Nancy Pelosi, a West Coast American from California, intended, one is inclined to believe, to force Biden, from Delaware on the East Coast, to follow in the footsteps of Obama, hailing from Hawaii, by returning to his “pivot to Asia” and letting Russia be Russia.

Would Europe then get its act together? Much would depend, it seems, on France and how it will try to achieve Macron’s “European strategic sovereignty”, recently renamed “strategic autonomy” by Scholz in order not to draw even more ire from his American friends. Call it sovereignty, call it autonomy, possible it would be only together with Germany. Lacking nuclear arms, however, Germany still considers itself dependent for its national security on the USA, which maintains about 30,000 troops on German soil, alongside an untold number of nuclear warheads. Will France be willing, in the name of European strategic sovereignty, to Europeanize its nuclear force, and with it its seat on the UN Security Council, thereby also Germanizing the two? What kind of constitution, or de facto constitution, would “Europe” require for France to bequest to it its remaining great power *accoutrements*, for the sake of building an even greater European power together with Germany? It would be useful if in his next book, van Middelaar did not apply his impressive inside knowledge merely to painting in bright colors and making us enthusiastically wait for the happy hour when a new European event-politics will somehow take command, and with it a great European leader, a Churchill or de Gaulle, or another Merkel whom van Middelaar strangely enough sees in the same league as the two others. What we need instead is a sober analysis of the structural conditions, restrictive as well as conducive, and of the realistically possible organizational forms for a Europe capable of keeping its distance from both China and the USA, with a sustainably de-globalized economy and a credible security architecture that, hopefully, will protect it from being drawn into the impending bloody battle between a declining and a rising global hegemon.

## Footnotes

1 *Quand l’Europe improvise: dix ans de crises politiques* (2016: Gallimard, Paris)

2 The Schengen Area is made up of twenty six European countries that have eliminated all passport and all other types of border control at their mutual borders. The area is named after the 1985 Schengen Agreement and the 1990 Schengen Convention, both ratified in Schengen, Luxembourg.

